# Comparison of Meconium Microbiome in Dizygotic and Monozygotic Twins Born by Caesarean Section (CS)

**DOI:** 10.3389/fmicb.2020.01139

**Published:** 2020-06-03

**Authors:** Jing Yang, Su Yao, Kun Cheng, Lili Xu, Lingling Hou, Yuan Wei, Huijun Feng, Xuejian Yu, Xin Zhang, Xiaomei Tong, Zailing Li, Yangyu Zhao

**Affiliations:** ^1^Department of Obstetrics and Gynecology, Peking University Third Hospital, Beijing, China; ^2^China Center of Industrial Culture Collection (CICC), China National Research Institute of Food & Fermentation Industries Co., Ltd., Beijing, China; ^3^Department of Pediatrics, Peking University Third Hospital, Beijing, China; ^4^Computational Genomics Lab, Beijing Institutes of Life Science, Chinese Academy of Sciences, Beijing, China

**Keywords:** meconium microbiome, dizygotic twins, monozygotic twins, genetics, 16S rRNA gene sequencing

## Abstract

The early-life microbiota triggers life-long effects on physiological functions and health disorders. Previous studies in adult twins or animal models have revealed associations between host genetics and the harmonious microbiota. However, such associations may be obscured by the fact that each intra-pair of twins will continually encounter various environmental factors as they grow up. Here, we collected the meconium samples from nineteen dizygotic pairs (DZ, *n* = 38) and nine monozygotic pairs (MZ, *n* = 18) with cesarean delivery, and 16S rRNA gene sequencing was performed to profile the microbiome at birth. Diversity analysis showed that alpha diversity was not significantly different between two groups, whereas beta diversity of MZ twins was significantly lower than that of either DZ twins or unrelated individuals (i.e., randomly selected individual pairs of non-twinship) (*p* < 0.05). Two groups had very similar microbial classifications but different relative abundances of certain taxa including more Firmicutes (*p* = 0.05, Wilcoxon test) at the phylum level and lower abundances of five genera (*p* < 0.05) in DZ group compared to MZ group, including *Rheinheimera*, *Proteus*, *SMB53*, *Sphingobium*, and *Megamonas*. Co-occurrence analysis in each group showed slightly more complicated microbial interactions in DZ than MZ twins, although 22 shared bacterial genera co-existed in two groups, with both *Rheinheimera* and *Megamonas* having different centralities in their respective co-occurrence networks. Mean intra-class correlation coefficient (ICC) were also significantly higher for MZ (0.312) compared to DZ twins (0.138) (*p* < 0.05). The predicted microbial gene functions related to carbohydrate were higher in DZ group, whereas folding, sorting, degradation, cell motility pathways and energy metabolism were markedly over-represented in the microbiota of MZ group. In summary, our study uncovered that microbial diversity and components of the meconium microbiome between DZ and MZ twins were partially consistent with that in singleton neonates by cesarean delivery, but several distinctions related to the heritability supported genetic contributions to intestinal microbiome in early life.

## Introduction

Growing studies have documented microbiota of newborn meconium which influenced immune and metabolic development in early-life and might, therefore, have short and long-term health consequences (Marie-Claire et al., 2014; Tamburini et al., 2016; Milani et al., 2017). Aberrant bacterial communities can lead to disease through an altered development of the immune system (O’Toole and Claesson, 2010; Wilczynska et al., 2019). Based on results from large-scale human population and mouse studies under controlled conditions, genetics is an indispensable shaper of microbial diversity and composition related to diet, innate immunity, and energy metabolism etc. (Dicksved et al., 2008; Smith et al., 2013; Li et al., 2014). Therefore, it is urgent to better explore how genetics factors contribute to early-life microbiome composition.

Twins, with their high genetic and environmental resemblance, are well appropriate for probing the role of the gut microbiome and clarifying gene-environment effects on complex phenotypes. Each intra-pair of monozygotic (MZ) and dizygotic (DZ) twins share same environment during pregnancy, but their genetic similarity is different: 100% in MZ intra-pair similarity twins versus, in general, 50% in DZ twins. If host genetics may conceivably influence a particular microbiological phenotype, there are more similar phenotype within MZ compared to within DZ twins.

Current twin studies have not only identified more heritable bacteria (Dicksved et al., 2008) but also verified the importance of host genetics in determining gut microbiome composition (Smith et al., 2013). Nevertheless, the subject of former twin studies was adults with a mean age of 61 years (36–80 years of age) and their phenotypes were not selected and further compared. Decreased concordance of the gut microbiome between MZ and DZ twins was affected by many factors such as sharing of household, geographic region (Li et al., 2014), individual diet, diseases, microbiome-based forensics and disease treatments (Ridaura et al., 2013; Franzosa et al., 2015) and so on, which may attenuate the reliability of the former results (Smith et al., 2013). Therefore, meconium which provides the individual-specific information about initial microbiome composition may be a more suitable material for exploring the effect of the genetics on microbiome (Wilczynska et al., 2019).

Here, we performed 16S rRNA gene sequencing on the earliest in life stools of twin neonates including 9 pairs of MZ twins and 19 pairs of DZ twins. This study demonstrated the characteristics of meconium microbiome in neonatal twins. Combining rigorous analytical methods, similarities and differences of the intestinal microbiome between MZ and DZ twins were comprehensively clarified, thereby providing a knowledge base for the genetic contribution to optimal microbial communities in early life.

## Materials and Methods

### Subject Details

Written informed consent was obtained from the pregnant women with gestation age ≥34 weeks (*n* = 28) enrolled at hospital admission for their non-emergency cesarean section at Peking University Third Hospital in China with the approval of the Human Research Ethics Committee of this hospital (IRB00006761-2016145). Maternal and fetal characteristics were obtained from electronic medical records and questionnaire surveys. Inclusion criteria for mothers were: a twin pregnancy of known chronicity, definite pregnancy outcome, delivery of 2 live fetuses, and information available on maternal height and weight, parity, and ethnicity, accuracy of gestational age. Exclusion criteria contained: at least 1 fetal structural or chromosomal anomalies; monochorionic-monoamniotic twins; complicated monochorionic twin pregnancies including twin-to-twin transfusion syndrome (TTTS), twin reversed arterial perfusion (TRAP), selective intrauterine growth restriction (sIUGR), twin anemia-polycythemia sequence (TAPS); a history of fetal therapy; infants who received postnatal antibiotics or whose mothers used antibiotics within 3 months before cesarean delivery; twins with intra pair birth weight difference ≥25%; maternal complications (e.g., hypertensive disease, diabetes, or autoimmune disease); neonatal complications (e.g., neonatal necrotizing enteritis, neonatal asphyxia, etc.).

### Sample Collection

First-pass meconium samples were collected within the first 24 h of delivery (mean: 12.33 h; range: 0.67–14.6 h). Among 28 twin pairs, 8 twin pairs were discordant for meconium collection before or after receiving colostrums, which meconium in one twin was sampled after first breastfeeding and meconium samples in the other twin were obtained before first breastfeeding. Twenty twin pairs were concordant for meconium collection before receiving colostrums. Sampling operations were performed by trained professionals under a uniform protocol. Approximately 2–5 g fecal samples were collected from sterile single-use diapers into sterile Sarstedt feces tubes (No. 80.734.311, Germany) with DNA stabilizer inside. All samples were mixed up thoroughly with the DNA stabilizer and stored at −80°C within 6 h of collection until total DNA extraction for later sequencing.

### DNA Extraction

In a strictly controlled and sterile laboratory, about 0.2 g of each fecal sample was processed using the QIAamp DNA Stool Mini Kit (QIAGEN, Hilden, Germany) for DNA extraction. The manufacture’s instruction was followed with the modifications being that, the vortex step was replaced with vertical shaking with oscillation frequencies of 50 Hz for 2 min using the TissueLyser LT instrument (QIAGEN), all the lysate mixture instead of specified 200 μL was used for subsequent extraction, and the ultimate column was eluted twice with the same amount of distilled water. The DNA concentration and molecular size were measured by using Qubit 3.0 fluorometer (Life Technology, Waltham, MA, United States) and by agarose gel electrophoresis, respectively.

### PCR and High-Throughput Sequencing

For each fecal sample, we amplified V4 variable region of the 16S rRNA gene by PCR using 515F (5′-GTGCCAGCMGCCGCGGTAA-3′) and 806R (5′-GGACTACHVGGGTWTCTAAT-3′) primers with VeraSeq 2.0 DNA Polymerase (Enzymatics) for 30 cycles. Positive amplicons were purified with Agencourt AMPure XP Beads (AGENCOURT, No. A63882), pooled with different index sequences and 2 × 250 bp paired-end (PE) sequenced on the Illumina HiSeq 2500 sequencing platform.

### 16S rRNA Gene Sequence Analysis

Raw sequencing reads were filtered with the following exclusion: with N bases, with contamination of adapters, with less than 75% high-quality bases (Phred score ≥ 20) or with low complexity (a certain base appears continuously for 10 times). PE reads were merged into single tags by overlapping with FLASH (Magoc and Salzberg, 2011). Operational taxonomic units (OTU) were formed from merged tags and clustered using UPARSE (Edgar, 2013) with a sequence similarity cut-off of 97%. OTUs were taxonomically classified with the Greengenes 16S rRNA gene database V201305 (Mcdonald et al., 2012). Alpha and beta diversities and the rarefaction curves were calculated in Mothur v1.31.2 (Schloss et al., 2009) and QIIME v1.80 (Caporaso et al., 2010), respectively. The taxonomic composition of bacterial communities, multivariate ANOVA based on dissimilarities (Adonis), principal component analysis (PCA), and the heatmap clustering on the genus taxonomic level were analyzed in R packages gplots, vegan, ade4, and gplots, respectively. Linear discriminant analysis (LDA) Effect Size (LEfSe) analyses were performed with the software LEfSe to identify potential microbial biomarkers between groups (Segata et al., 2011). By virtue of PICRUSt v1.0.0 (Langille et al., 2013), functional prediction was made on the abundance of KEGG pathways from the 16S rRNA gene data. Intraclass correlation coefficients (ICCs) were generated using the SPSS statistical software (version 20).

### Co-occurrence Network Analysis of the Microbiome in DZ vs. MZ Twin Groups

To address microbial interactions in each group of DZ twin and MZ twin, we performed a co-occurrence network analysis for gut bacteria in each group. The bacterial correlations of each group were computed, respectively, based on the relative abundance of each genus using SparCC (Friedman and Alm, 2012) with 100 bootstraps to estimate the *p*-value. The correlation values with *p*-value < 0.05 and correlation values *r* > 0.9 were retained.

## Results

### Subjects and Sequencing Data Characteristics

A total of 56 samples of the first stool (meconium) from 28 twin pregnancies including 9 MZ twin pairs and 19 DZ twin pairs were collected. Baseline demographic, maternal and neonatal outcomes characteristics were displayed in Tables 1, 2 including pre-pregnancy BMI (Body Mass Index), gestational weight gain of mothers and birth weight, etc. There was no statistic difference in either maternal or neonatal characteristics between two groups, except for significantly higher proportion of natural conception in MZ twins (*p* < 0.001^∗∗^).

**TABLE 1:** Maternal characteristics for the study cohort.

**Variables**	**MZ twin group (*n* = 9)**	**DZ twin group (*n* = 19)**	***p*-value**
Maternal age (years)^a^	32.0 ± 3.9	32.5 ± 4.3	0.782
Maternal pre-pregnancy BMI (kg/m^2^)^a^	22.0 ± 3.4	21.7 ± 4.3	0.796
Weight gain during pregnancy (kg)^a^	17.9 ± 7.4	18.6 ± 4.5	0.767
**Primigravida (%)**			
Yes	66.7	78.9	0.646
No	33.3	21.1	
**Han ethnicity (%)**			
Yes	100.0	94.7	1.0
No	0.0	5.3	
**Employment (%)**			
Yes	88.9	100.0	0.321
No	11.1	0.0	
**IVF-ET/Natural conception (%)**			
IVF-ET	0.0	84.2	<0.001**
Natural conception	100.0	15.8	

^*a*^Data are given as mean ± standard deviation. IVF-ET, In Vitro fertilization-embryo transfer. BMI, Body Mass Index. **p < 0.01.

**TABLE 2:** Characteristics of neonatal outcomes (*n* = 56).

**Variables**	**MZ twins (*n* = 18)**	**DZ twins (*n* = 38)**	***p*-value**
Gestational age at delivery (weeks)^a^	36.11.7	36.31.2	0.613
Birth weight (g)^a^	2513.9323.6	2571.6302.9	0.518
Umbilical blood pH^a^	7.350.05	7.410.0	0.857
Height (cm)^a^	45.62.2	46.11.9	0.451
Abdominal circumference (cm)^a^	31.31.4	31.81.3	0.214
Head circumference (cm)^a^	33.21.1	33.31.2	0.843
Apgar score at 1 min^a^	9.90.2	10.00.0	0.049*
**Gender (%)**			
Male	49.9	52.6	0.567
Female	50.1	47.4	

^*a*^Data are given as mean ± standard deviation. *p < 0.05.

The 16S rRNA gene sequencing of 56 fecal samples yielded 3,430,164 clean PE reads (2 × 250 bp), with ∼122,506 reads per sample (Supplementary Table S1). More than 99.4% of the PE reads were successfully merged into single sequences according to overlaps. Good’s coverage and rarefaction curve estimation were performed with the sequenced data (Supplementary Figure S1) and the results showed that the number of sequences can well represent the diversity of each microbial community and thus can support our subsequent analyses.

### Microbial Community Structure of the Meconium in Twins

**FIGURE 1:**
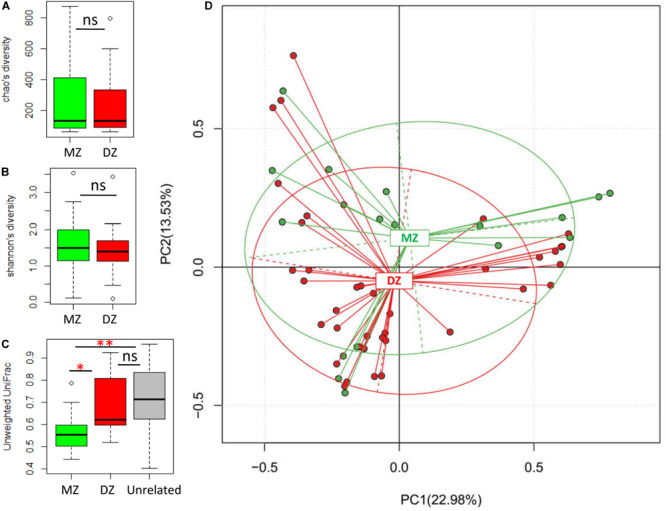
Microbial comparisons between DZ and MZ twins. **(A,B)** Alpha diversities of DZ and MZ using the Chao and Shannon indices. **(C)** Unweighted UniFrac distances between microbial communities obtained by comparing individuals within twinships for DZ and MZ twin pairs, and between unrelated individuals (Unrelated). **(D)** Principal component analysis (PCA) plot based on the relative taxa abundance in the microbiomes of DZ and MZ neonates. ns, not significant. *p < 0.05, **p < 0.01.

We performed alpha diversity assessments between DZ and MZ twin pairs by Chao and Shannon indices (Figures 1A,B). Although lower microbial diversity in DZ than MZ groups, neither of those indices were significantly different between two groups. This demonstrated that the meconium microbiome exhibited similar species richness and evenness in two groups, which may be considered as consistent intestinal homeostasis in normal twin neonates.

The index of unweighted and weighted UniFrac distance was used to assess the beta diversity between the twin pairs. The beta diversity based on unweighted UniFrac between MZ twin pairs was significantly lower than that between DZ twin pairs (*p* < 0.01^∗∗^; Figure 1C), whereas there was no significant difference in community distance between DZ and unrelated pairs (i.e., randomly selected individual pairs of non-twinship). The results revealed that MZ twin pairs were significantly more similar to each other in their microbiome than either DZ twin pairs or unrelated individuals. The beta diversity based on weighted UniFrac between MZ twins was also significantly lower than that of unrelated pairs (*p* < 0.05^∗^, Supplementary Figure S2). To further observe the clustering effect of microbial communities between MZ and DZ twins, PCA was carried out (Figure 1D). The results showed that gut microbiota from two groups were slightly gathered into two clusters despite no statistical significance in Adonis analysis (*R*^2^ = 0.00295, *p* = 0.881).

### Global Taxonomic Features of the Microbial Community

**FIGURE 2:**
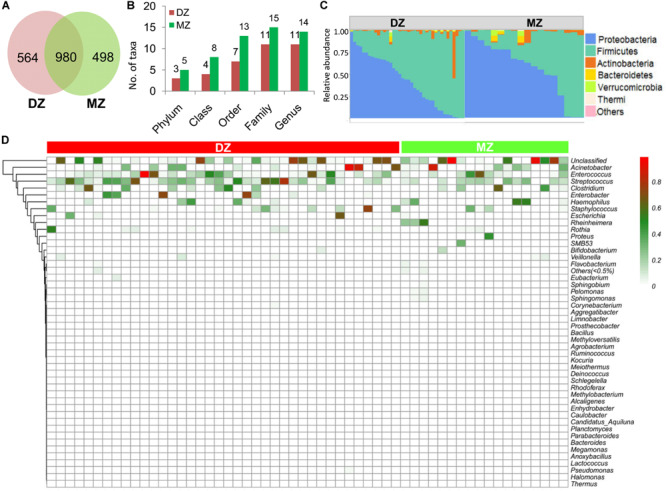
Analysis of OTUs and microbial composition in DZ and MZ. **(A)** Numbers of unique and shared operational taxonomical units (OTUs) between DZ and MZ. **(B)** Numbers of phylum, class, order, family, and genus that the OTUs were classified into in DZ and MZ. **(C)** Relative abundance of bacterial phyla in DZ and MZ. **(D)** Heatmap showing relative abundances of 48 genera (>0.5%) between DZ and MZ. The bar represents the proportion of OTUs affiliated with the genera.

Among 2,042 OTUs, 498 and 564 were unique in MZ and DZ, respectively, and 980 were shared by the two groups (Figure 2A). The results of classification and annotation at the phylum, class, order, family and genus levels for all samples were shown in Figure 2B. By evaluating the average relative abundance, we detected 5 and 3 phyla, 8 and 4 classes, 13 and 7 orders, 15 and 11 families, 14 and 11 genera across all of samples from MZ and DZ twins, respectively. The numbers between two groups were similar at each classification level. At the phylum level, Proteobacteria (41.90 and 56.88% of total bacteria in DZ twins and MZ twins, respectively) and Firmicutes (54.08% in DZ twins, and 38.58% in MZ twins) were two dominant phyla, totally being prevalent and representing over 95% of the microbiota in either group. For relative abundance of bacterial phyla, more Firmicutes (*p* = 0.05, Wilcoxon test) was found in DZ group compared to MZ group. Lower abundance of Actinobacteria accounted for 3.15% (DZ twins) and 2.20% (MZ twins), followed by Bacteroidetes comprising 0.33% (DZ twins) and 0.94% (MZ twins) (Figure 2C).

At the genus level (Figure 2D), the most dominant bacteria (average relative abundance >1%) were as follows: *Streptococcus* (22.67 and 11.17% in DZ and MZ twin pairs, respectively), *Enterococcus* (15.75 and 11.14%), *Acinetobacter* (10.53 and 11.28%), *Clostridium* (7.26 and 8.38%), *Staphylococcus* (6.05 and 3.35%), *Haemophilus* (3.34 and 8.69%), *Enterobacter* (6.90 and 0.05%), *Rothia* (2.77 and 1.01%), *Escherichia* (2.79 and 0.22%) and *Rheinheimera* (0.02 and 5.72%). Most of them were widely shared, such as certain opportunistic pathogen *Acinetobacter*. In addition, *Bifidobacterium*, one of the common and key adult gut residents, was observed in 73.2% of all the samples, despite being in a relatively low abundance in DZ (0.18%) and in MZ (0.99%).

### The Differences in the Relative Abundance of Bacteria Between Two Groups

**FIGURE 3:**
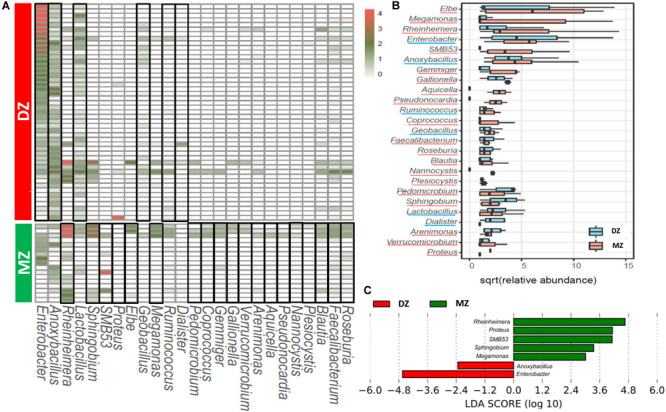
Heatmap **(A)** and box-plot **(B)** of the 25 genera with significant different abundance between DZ and MZ. **(C)** LEfSe analysis between DZ and MZ showing genera with LDA score absolute values > 2.0. Genera that were enriched in DZ or MZ groups were framed in black in the heatmap **(A)** and they were underlined in different colors in the box-plot **(B)**.

The LEfSe analysis was performed for the exploration of relative taxa abundance, characterized by significant differences between two groups. Among 25 genera with different relative abundance (Figures 3A,B), seven genera had the absolute value of log_10_ (LDA score) > 2.0 (Figure 3C). Comparing DZ with MZ twin pairs, the former included more *Enterobacter* (6.90% vs. 0.05%, *p* < 0.01^∗∗^) and *Anoxybacillus* (0.07% vs. 0.03%, *p* < 0.05^∗^) and the latter over represented five genera including *Rheinheimera* (0.02% vs. 5.72%, *p* < 0.01^∗∗^), *Proteus* (0 vs. 2.53%, *p* < 0.01^∗∗^), SMB53 (0 vs. 1.80%, *p* < 0.01^∗∗^), *Sphingobium* (0 vs. 0.29%, *p* < 0.01^∗∗^), and *Megamonas* (0 vs. 0.04%, *p* < 0.05^∗^). These genera might represent candidates for microbial biomarkers between the two kinds of neonate twins. Microorganisms including *Enterobacter*, *Anoxybacillus*, and *Proteus* have been reported to be important for host health, and aberrant changes in their abundances may result in various human gut diseases (Gerritsen et al., 2011; Geng et al., 2013; Tamburini et al., 2016; Martens et al., 2018).

**FIGURE 4:**
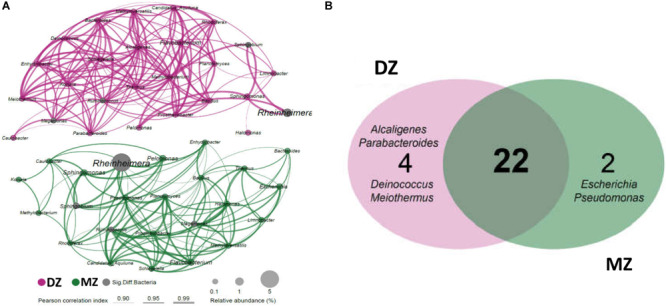
Bacterial co-occurrence networks of DZ and MZ pairs. **(A)** Each node in the network indicates a bacterial genus. Node size represents the average relative abundance of one genus in each twins group. The thickness of edges represents the correlation value. Only the bacterial connections (edges) larger than cut-offs (correlation value *r* > 0.9, *p* < 0.05) were shown. **(B)** Shared and unique genera between networks of the two groups. Twenty-two genera present in at least 50% of all samples of each group were shared by the two groups, and 2 and 4 genera were unique in MZ and DZ pairs, respectively.

For intra-traits of the meconium microbiome in two groups, co-occurrence networks were constructed aiming to clearly dissect the species interconnections within the structure and capture the information about important community characteristics. The network topology was slightly more complicated in DZ twin than MZ twin pairs (Figure 4A). Totally 26 genera (nodes) were strongly correlated (SparCC, *r* > 0.9, *p* < 0.05) in the co-occurrence network of DZ twin pairs, while 24 genera (nodes) were presented in that of MZ pairs. The network density of DZ and MZ was 45.85% (149 edges) and 42.39% (117 edges), respectively. In the networks, 22 genera including *Rheinheimera*, *Ruminococcus*, *Bacteroides*, and *Bacillus* etc. kept stable with strong positive correlation with others in both MZ and DZ groups. Two genera of *Rheinheimera* (EigenCentrality, 0.005 in DZ and 0.721 in MZ) and *Megamonas* (EigenCentrality, 0.722 in DZ and 0.871 in MZ) with significantly higher relative abundances in MZ twin pairs, had different centralities in their respective co-occurrence networks, which were related to different taxa and numbers of genera in MZ and DZ co-occurrence networks. Four genera including *Alcaligenes*, *Parabacteroides*, *Deinococcus*, and *Meiothermus* were unique for DZ twin pairs and two genera including *Escherichia* and *Pseudomonas* were unique for MZ twin pairs (Figure 4B). The shared 22 genera may adapt to the same niche and tend to co-occur in a positively related network module indicating similar bacterial interactions between two groups. However, relatively simpler bacterial co-occurrence networks in MZ than DZ twins may support genetic influences on the gut microbiome in early life.

**FIGURE 5:**
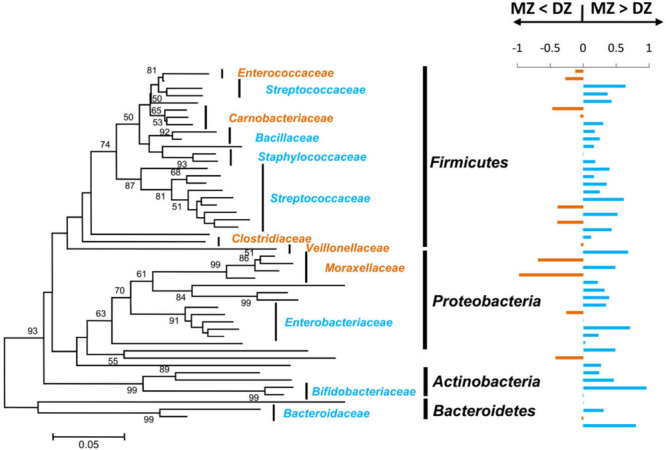
Operational taxonomic units relative abundances correlated within MZ and DZ twin pairs. On the left is a neighbor-joining phylogenetic tree of OTU shared by >50% of the samples in this study. Major bacterial families that the OTUs were classified into were labeled. Bootstrap values > 50% were shown and the bar shows 5% difference in the nucleotide sequence. On the right are corresponding twin-pair intraclass correlation coefficients (ICCs). For each of the OTUs, ICCs were calculated and compared between DZ and MZ. Bars that point to the right represent that the difference is positive (i.e., MZ ICCs > DZ ICCs), colored in blue, and bars that point to the left represent negative differences (i.e., DZ ICCs > MZ ICCs), colored in orange. The families were colored in accordance with the bars.

For each twin pair, we generated ICCs for the relative abundances of OTUs shared by >50% of the samples (Figure 5). Based on the OTUs-generated ICCs, a constructed phylogenetic tree fell into multiple bacterial families, including Streptococcaceae, Enterobacteriaceae, Staphylococcaceae, Bacillaceae, Bifidobacteriaceae, and Bacteroidaceae. The majority (72.9%) of the OTUs had higher ICC values within MZ than DZ pairs, and mean ICCs were significantly greater for MZ (0.312) compared to DZ pairs (0.138) (*p* < 0.01^∗∗^). High ICCs in MZ twin pairs clearly indicated that certain gut microbes within MZ twin pairs were more similar and highly correlated in abundance than within DZ twin pairs. These results provided evidence that the effect of the genetics on the gut microbiome composition in MZ group was stronger than in DZ group.

### Different KEGG Pathways Between DZ and MZ Twin Pairs

**FIGURE 6:**
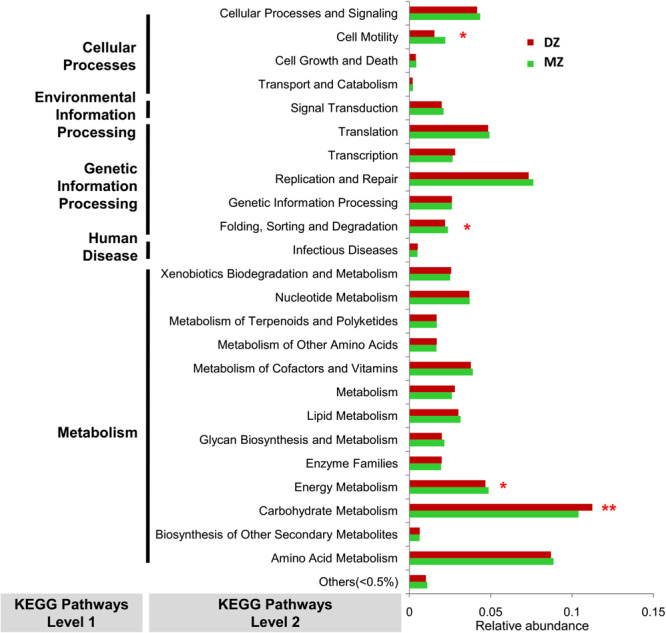
Variation in abundances of predicted KEGG pathways between DZ and MZ. *p < 0.05, **p < 0.01.

PICRUST analysis revealed that genes were mainly assigned to five KEGG categories on level 1: cellular processes (abundance of genes involved were 2.15 and 2.86% in DZ and MZ pairs, respectively), environmental information processing (18.82 and 17.67%), genetic information processing (17.17 and 17.56%), human diseases (0.87 and 0.90%) and metabolism (45.63 and 45.45%). Results from a Student’s *t*-test revealed that the majority of the KEGG pathways on level 2 (84.0%, 21/25) were not significantly different in abundance between DZ and MZ pairs. The pathway of carbohydrate metabolism was enriched in DZ pairs (*p* < 0.01^∗∗^). Relatively, genes in MZ pairs seemed to be overrepresented in pathways of energy metabolism, cell motility and folding, sorting and degradation (*p* < 0.05^∗^) (Figure 6).

## Discussion

Evidence of host genetic control of microbiome using high-throughput sequencing approaches is scarcer at the early life in the context of the twin design. Different from the existing studies (Guo et al., 2016; Tang et al., 2018), first-pass twins meconium samples were taken and analyzed for teasing inevitable environmental factors in this study. Our study reported the inter-characteristics and intra-traits of meconium microbiome in different chorionic twin pregnancies evaluated by microbial diversity, taxa abundance, individual microbial components and bacterial interactions. In spite of no difference in the alpha diversity, taxonomic composition and corresponding proportion of the bacterial microbiome were obviously different between DZ and MZ groups. Previous studies concluded that individuals living together have more similar microbial populations (Stahringer et al., 2012; Song et al., 2013). In this study, both twin types share the same intrauterine environment, and heritability of traits can be directly estimated. Co-twins in MZ twins show more similarity in their microbiome than DZ twins based on higher ICCs, lower beta diversity and simpler network topology in MZ twin pairs. These results may be responsible for human genetic influences on the microbiome at the stage of the earliest life.

This study may establish an important baseline regarding the features of the twin pregnancies neonatal gut microbiota partly similar to that of singleton pregnancy by C-section delivery. The bacteria we found in the meconium of twins are consistent with the findings of many cultured and culture-independent studies. Twin neonates born by C-section are colonized by dominance of four phyla including Proteobacteria, Firmicutes, Actinobacteria, and Bacteroidetes. This result was consistent with a previous study aiming to characterize the structure of intestinal microbiome community in very low birth weight infants with extrauterine growth restriction (Li et al., 2019) and infant twin pairs (Maqsood et al., 2019), in which the main phyla were the four phyla and Proteobacteria was the most abundant phylum among them. It was also reported in former studies that Proteobacteria and Firmicutes were the main phyla represented during the first days of life in the feces of C-section-delivered (Jakobsson et al., 2014). However, despite having similar microbial classifications, the abundance of Firmicutes (54.08%) and Firmicutes/Bacteroidetes ratio in DZ twins were higher than those in MZ twins. This results were unachievable in previous studies on the gut microbiome of singleton neonate and adult twins, which may be affected by a variety of maternal factors (e.g., the way of conception and delivery), or different rules of the inter-generation transmissibility from mothers to babies at birth, infant gender, hospitalization after birth, etc. (Di Gioia et al., 2014; Robertson et al., 2019). In our study, the dominant genera were *Streptococcus*, *Enterococcus*, *Acinetobacter*, *Clostridium*, and *Staphylococcus* with unanimous results of recent microbiome studies (Collado et al., 2010; Younge et al., 2019). Cultivatable bacteria belonging to the genera *Enterococcus*, *Streptococcus*, and *Staphylococcus* have been isolated from nearly half of the umbilical cord and from all of the meconium samples in some previous studies (Jimenez et al., 2005; Jiménez et al., 2008). *Staphylococcus* and *Streptococcus* were the characteristic of initial colonization of the intestinal microbiota in C-section infants (Dominguez-Bello et al., 2010, 2016). A birth cohort study conducted in three European countries confirmed high prevalence of Clostridia in C-section-delivered infants compared with vaginally delivered (Adlerberth et al., 2007), which was accordant with the India’ study (Pandey et al., 2012). In addition, twin infants less colonized with *Bifidobacterium* and *Bacteroides* genera similar to the singleton (Azad et al., 2013). These results demonstrate the accuracy of the bacteria we identified in the meconium sample. LEfSe analysis found 7 differentially abundant taxa between two groups, and these taxa may be potential biomarkers.

The results of higher ICCs in MZ than in DZ twin pairs have been previously observed either in the gut microbiome of adults (Goodrich et al., 2016) or in the oral microbiome (Demmitt et al., 2017). In addition, the OTUs with the higher correlation in MZ twins fell into six bacterial families, including Streptococcaceae, Enterobacteriaceae, Staphylococcaceae, Bacillaceae, Bifidobacteriaceae, and Bacteroidaceae in this study. Consistently, ICC values for Streptococcaceae, Bacteroidaceae, and Bacillaceae are were significantly greater in MZ than DZ pairs in oral microbiome, and the stability of which over time in adults was reported to be remarkably high relative to that of other body sites (Lazarevic et al., 2010; Caporaso et al., 2011; Consortium, 2012; Cameron et al., 2015). These results may indicate that certain microbes had indeed intra-pair correlationsin abundance within MZ than DZ pairs free of the environment, behavioral, and cohabitational effects on the microbiome. Furthermore, *Bifidobacterium* genus was highly heritable bacteria in former studies such as the twins in UK population (Goodrich et al., 2016), the Human Microbiome Project (HMP) (Blekhman et al., 2015), the Hutterites (Davenport et al., 2015), and mouse studies (Benson et al., 2010; Leamy et al., 2014), which is in accordance with the results of our study.

Previous studies have shown that the Firmicutes phylum ferment unabsorbed carbohydrates and the presence of this bacterium with high concentrations in children can lead to increased energy harvesting from unabsorbed carbohydrates. The Firmicutes/Bacteroidetes ratio plays an important role in regulating host energy metabolism and an elevated Firmicutes/Bacteroidetes ratio demonstrated the effective energy intake mediated by the intestinal microbiota (Jumpertz et al., 2011). In this study, the microbial gene functions related to carbohydrate metabolism were higher in the fecal microbiome of DZ twins, which may partly ascribe to higher abundance of Firmicutes and Firmicutes/Bacteroidetes ratio. Carbohydrate associated genes were mapped to the Enterobacteriaceae in the study of the intestinal microbiota community of neonatal necrotizing enterocolitis (Claud et al., 2013). Consequently, higher abundance of the genus *Enterobacter* in DZ group may be related to up-regulation of pathways involved in carbohydrate metabolism in DZ twins. In previous study (Polansky et al., 2015), the species *Megamonas* spp. involved in cholesterol metabolism (Maya-Lucas et al., 2019) were over-represented in obese children. A significantly higher abundance of the *Megamonas* genus in MZ group might imply up-regulation of pathways involved in lipid and energy metabolism in MZ twins.

## Conclusion

This study provided landscape of meconium microbiomein both MZ and DZ twins born by caesarean section (CS). Simpler bacterial co-occurrence networks, lower beta diversity and more genera with high ICCs in MZ twin pairs supported genetic determinants of meconium microbiome in view of higher microbiome similarity between co-twins in MZ groups. Considering that meconium optimally keeps the concordance of the gut microbiome between MZ and DZ twins, genetic contributions to the gut microbiome were convincingly supported. The limitations in this study were relatively small sample size and the difference of conception ways between two groups. However, there is consensus in the literature that the conception by *In Vitro* Fertilization-Embryo Transfer (IVF-ET) does not increase the rate of neonatal adverse outcomes. There was no definite conclusion on higher incidences of maternal complications. In only one study (Shi et al., 2018) on the meconium microbiome of two newborns by IVF-ET conception delivered via cesarean section, the result failed to support different modes of conception affected microbiome compositions. In addition, strict inclusion and exclusion criteria greatly reduced maternal and fetal influence on meconium microbiota ensuring accurate and differential results between two groups. Future work will focus on the following studies that heritable components of the meconium microbiome should be identified and further discern to what extent the gut microbiome is determined by human genetics partly by longitudinal and dynamic follow-up.

## Data Availability Statement

Raw sequences reads generated in this study have been deposited in GenBank database under accession number PRJNA530088. The confirming data and crucial materials during the current study are available from the corresponding author on rational request.

## Ethics Statement

Written informed consent was obtained from the pregnant women with gestation age ≥34 weeks (*n* = 28) enrolled at hospital admission for their non-emergency cesarean section at Peking University Third Hospital in China with the approval of the Human Research Ethics Committee of this hospital (IRB00006761-2016145).

## Author Contributions

YZ, SY, and YW conceived this study. JY, XT, ZL, and LX recruited participants and collected samples. JY wrote the manuscript and prepared the figures with help from KC. All authors provided critical intellectual content and approved the final manuscript.

## Conflict of Interest

SY, KC, HF, XY, and XZ were employed by China National Research Institute of Food & Fermentation Industries Co., Ltd.

The remaining authors declare that the research was conducted in the absence of any commercial or financial relationships that could be construed as a potential conflict of interest.
