# Supplementary material for: Comparison of Meconium Microbiome in Dizygotic and Monozygotic Twins Born by Caesarean Section (CS)
Source: Front Microbiol. 2020 Jun 3;11:1139. doi: 10.3389/fmicb.2020.01139 (PMC7283445; doi:10.3389/fmicb.2020.01139)
Supplement: Supplementary file 1 [file Data_Sheet_1.docx]

**Supplementary Materials**

**Supplementary Figure S1**. Good’s coverage (A) and rarefaction curves based on multiple measures (B-E).


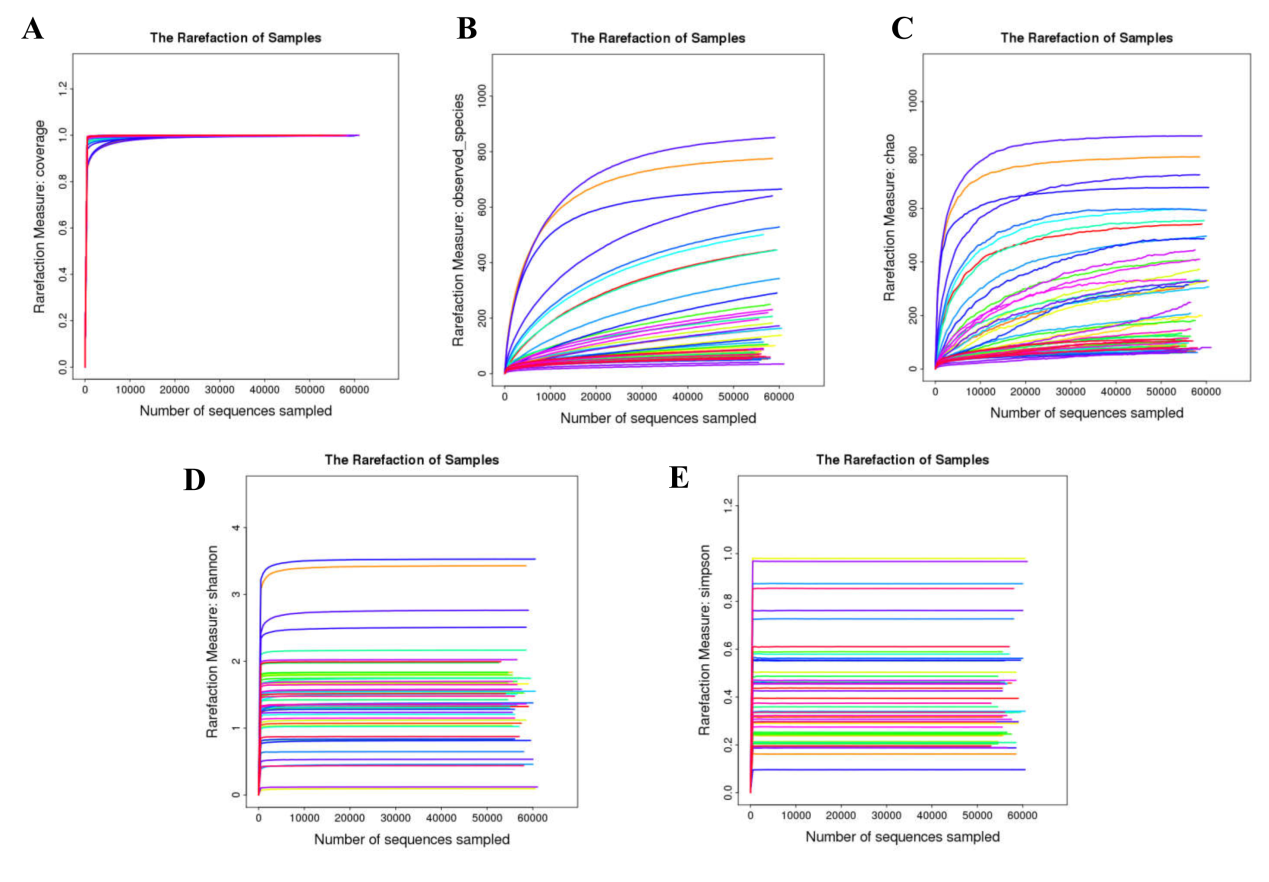


**Supplementary Figure S2**. Weighted UniFrac distances between microbial communities obtained by comparing individuals within twinships for DZ and MZ twin pairs, and between unrelated individuals (Unrelated).


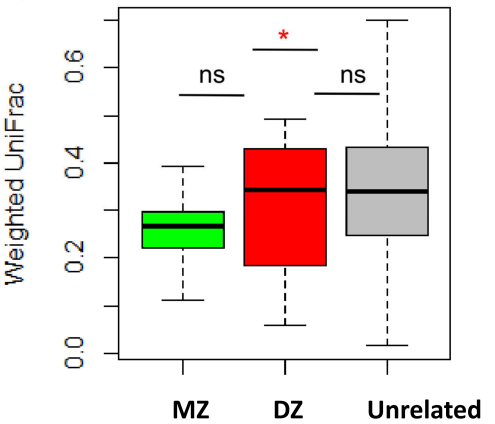


**Supplementary Table S1**. Sequencing data summary for all samples.
